# Cross-kingdom synthetic microbial communities outperform single-kingdom consortia in promoting potato growth under continuous cropping

**DOI:** 10.3389/fmicb.2026.1920336

**Published:** 2026-09-23

**Authors:** Wenqi Liang, Xu Ma, PingLiang Zhang, Wenming Zhang, Chenxu Yu

**Affiliations:** 1 College of Resources and Environmental Sciences, Gansu Agricultural University, Lanzhou, China; 2 Gansu Engineering Research Center for Resource Utilization of Livestock and Poultry Waste, Lanzhou, China; 3 Dry Land Agriculture Institute, Gansu Academy of Agricultural Sciences, Lanzhou, China; 4 Department of Agricultural and Biosystems Engineering, Iowa State University, Ames, IA, United States

**Keywords:** bacterial community, fungal community, pot experiment, potato, SynComs

## Abstract

Continuous cropping obstacles, including soilborne plant pathogen accumulation and soil microbial community degradation, present a critical bottleneck limiting sustainable potato production worldwide. To tackle the prominent obstacles associated with potato continuous cropping, this study explored the potential of synthetic microbial communities (SynComs) in regulating soil microbial ecology and promoting potato growth. Three SynComs were constructed, including a single bacterial consortium (SB), a single fungal consortium (SF), and a bacteria-fungal mixed consortium (SBF), using four bacterial strains (*Bacillus licheniformis*, *Streptomyces* sp., *Pseudomonas lundensis*, and *Microbacterium* sp.) and three fungal strains (*Penicillium* sp., *Aspergillus fumigatus*, and *Aspergillus terreus*). These SynComs were then evaluated for their effectiveness to overcome continuous cropping obstacles for potato in potted experiments, with non-inoculated group set as the blank control (CK). Our results demonstrated that the SBF consortium exhibited the most pronounced growth-promoting effect throughout the entire potato growth season. Specifically, SBF inoculation increased plant height, stem diameter, root length, and total biomass by 16.24%, 40.84%, 25.84%, and 13.86%, respectively, at the seedling stage; and 27.71%, 22.27%, 28.73%, and 39.31%, respectively, at the flowering stage. SynCom inoculation significantly altered both alpha and beta diversity of soil microbial communities and showed pronounced temporal dynamics. Mantel test analysis suggested that SynCom and pivotal bacterial taxa were the core driving factors for biomass accumulation in potato plants (*P* < 0.05). Collectively, SynComs effectively increased the abundance of inoculate-related microbes and restructured rhizosphere microbiota to promote potato growth. This study provides a sustainable strategy for alleviating potato continuous cropping obstacles and improving potato productivity.

## 1 Introduction

Potato (*Solanum tuberosum L*.) is a globally crucial staple crop, serving as a vital source of food, feed, and industrial raw materials. Owing to its unparalleled yield potential, strong resilience to abiotic stressors, and broad adaptability to diverse agroecological zones, it acts as a cornerstone crop for building climate-resilient food systems worldwide (Camire et al., 2009). Increasing high demand of potato promoted global expansion of potato cultivation and intensive cropping (Ju et al., 2006), which is often hindered by plant diseases and continuous cropping obstacle (Pédron et al., 2021). The assembly of rhizosphere microbiota could shape plant fitness and development by modulating nutrient acquisition, pathogen resistance, and phytohormone signaling networks (Falkowski et al., 2008; Hacquard et al., 2015), which have spurred innovation in microbial biotechnology for sustainable agriculture (Minty et al., 2013; Parnell et al., 2016). Rhizosphere microbiota harbors the richest microorganism reservoirs to underpin critical ecosystem function, such as nutrient cycling and pathogen suppression (Nemergut et al., 2013). To overcome the continuous cropping obstacles for crops such as potato, rhizosphere microbiota would play a key role if it could be properly manipulated.

Microbiome engineering is emerging as a transformative strategy for restructuring soil microbiota to enhance crop productivity. Targeted transplantation of synthetic microbial communities (SynComs) can reconfigure the composition and functional dynamics of soil microbial communities, directly suppressing phytopathogens while activating plant innate immunity and improving its nutrient-use efficiency (Trivedi et al., 2020). SynComs exhibit superior ecological resilience compared to monocultures in soil ecosystems, due to functional niche partitioning and cross-strain metabolic cooperation; these mechanisms collectively enhance biosynthetic capacity and resource use efficiency (Wang X. et al., 2019). Plant-microbe binary interaction assays, which are used to identify bacterial consortia with congruent host-modulating effects, represent a prospective method for the design of SynComs (Herrera Paredes et al., 2018). Proper plant-microbe assay can be designed to highlight the role played by plant root exudates, which have been shown to profoundly reshape soil microbial diversity by mediating plant-microbiota crosstalk (Yu et al., 2021; Zhalnina et al., 2018). Continuous cropping of potatoes changes the soil rhizosphere microbiota via selective pressure exerted by specific potato root exudates, favoring harmful microbes toward potato and leading to the occurrence of continuous cropping obstacles and the eventual reduction of the potato yield (Xing et al., 2023). In our previous research, we screened a microbial pool of 115 bacteria and 75 fungi which had the ability to degrade potato root exudates, and used the pool to make SynCom enhanced organic fertilizer to promote potato growth under continuous cropping (Zhang W. et al., 2024). To make effective SynCom in large scale industrial production, its composition needs to be simplified with limited number of microbial species for easier quality control and better batch-to-batch consistency. In this study, we aimed to produce SynComs with selected microbes that can be cultivated and maintained easily to assure production practicality. To do this, it is critical to find the key microbial species within the big pool we have identified, and construct SynCom communities with simpler compositions.

Therefore, in this study, seven microbial strains (four bacteria and three fungi) were selected that can grow fast and degrade potato root exudates with high efficiency, and then cross-kingdom bacterial-fungal SynComs were constructed. Each of these strains had unique functions; *Bacillus licheniformis* can inhibit the growth of pathogenic bacteria (Amrith et al., 2025), *Streptomyces* sp. can decompose organic matter and inhibit soil-borne pathogens, *Pseudomonas lundensis* can promote plant growth (Zhang W. et al., 2024), *Microbacterium spp*. can degrade hydrocarbons, produce indoleacetic acid, and solubilize inorganic phosphate (Sheng et al., 2009), *Penicillium* sp. can assimilate soluble organic matter (Zhang F. et al., 2024), *Aspergillus fumigatus* can accelerate the decomposition of lignocellulosic waste (Ezea et al., 2026), *Aspergillus terreus* is involved in soil carbon fixation (Alemayehu et al., 2024). SynComs constructed were then inoculated into soil in potted experiments of continuous cropping of potatoes to investigate their effects on potato growth and soil microbial community. We aimed to create simplified yet effective SynComs and provide an effective method to alleviate continuous cropping obstacles for potatoes.

## 2 Materials and methods

### 2.1 SynComs construction

4 bacterial strains (*Bacillus licheniformis*, *Streptomyces* sp. *Pseudomonas lundensis*, and *Microbacterium* sp.) and 3 fungal strains (*Penicillium* sp., *Aspergillus fumigatus*, and *Aspergillus terreus*) were selected from a pool of 115 bacterial strains and 75 fungal strains that have been shown to degrade potato root exudates (Zhang W. et al., 2024). These strains were selected due to their superior ability to degrade potato root exudates (Supplementary Table 1), and to grow rapidly. The 4 bacterial strains and 3 fungal strains were inoculated into Luria–Bertani (LB) broth and liquid sabouraud medium (SDB), respectively, and cultured at 28 °C with shaking at 150 rpm for 72 h. Same broths were used for bacteria (LB) and fungi (SDB), respectively, regardless of nutritional preference of individual species, mainly to simplify the cultivation operation so that potential large-scale industrial production would become easier to manage. Four bacterial strains (*Bacillus licheniformis*, *Streptomyces* sp., *Pseudomonas lundensis*, and *Microbacterium* sp.) and three fungal strains (*Penicillium* sp. *Aspergillus fumigatus*, and *Aspergillus terreus*) were then used to create three SynComs; for the synthetic bacterial communities (SB), equal volumes of the four bacterial cultures were mixed; for the synthetic fungal communities (SF), equal volumes of the three fungal cultures were mixed; and for the synthetic bacterial-fungal communities (SBF), equal volumes of all seven cultures were mixed. 200 mL of each SynCom were then used as one unit of treatment for each of the potted experiments.

### 2.2 Design of pot experiments of continuous cropping of potatoes

Topsoil samples (0–20 cm depth) were collected in 2024 from a 5-year continuously cropped potato field at the National Soil Quality Long-term Observation Station, Gansu Academy of Agricultural Sciences (104°36′ E, 35°35′ N). The local climatic conditions are as follows: an annual mean temperature of 6.2 °C, annual precipitation of 415 mm, annual evaporation of 1531 mm, altitude of 1970 m, and a frost-free period of 146–149 days. The basic soil physicochemical properties measured in 2024 were pH 8.07, electrical conductivity (EC) 0.63 mS.cm^–1^, total organic carbon 9.32 g.kg^–1^, total nitrogen 0.87 g.kg^–1^, available phosphorus 13.12 mg.kg^–1^, and available potassium 134.8 mg.kg^–1^.

After being air-dried, the soil was passed through a 1.0 cm sieve. Each pot was filled with 2.0 kg prepared soil. Basal fertilizer was incorporated at 0.1 g nitrogen per kilogram soil with an N/P ratio of 2:1; urea and potassium dihydrogen phosphate as nitrogen and phosphorus sources, respectively. The soil and fertilizer were evenly blended, followed by slow watering until water drained from pot bottoms. One virus-free potato mini-tuber was sown per pot and covered with 5 cm soil on the next day.

Four treatment groups were set up with ten replicates each, three replicates were randomly selected from five replicates at a time for each growth stage to be assessed/analyzed. Irrigation with different SynCom suspensions yielded four groups: SB, SF, SBF and CK. SB: 200 mL bacterial suspension + 200 mL blank fungal medium; SF: 200 mL fungal suspension + 200 mL blank bacterial medium; SBF: 200 mL mixed bacterial and fungal suspension (200 mL each); CK: equal volumes of blank bacterial and fungal medium (200 mL each). The blank bacterial and fungal media used for the CK treatment were incubated under the same conditions (28 °C, 150 rpm, 72 h) prior to application, to control for any potential effects of residual medium components on soil microbial communities. All plants were grown in the greenhouse of Gansu Agricultural University between June 2 and August 2, 2024.

### 2.3 Sample collection

Three pots were randomly selected for plant harvesting at the seedling stage (30 days after sowing, S) and flowering stage (60 days after sowing, F). Rhizosphere soil was fully homogenized and sampled via the quartering method. Each sample was split into three subsamples. Fresh soil was used to determine pH and electrical conductivity (EC). Air-dried samples were analyzed for soil organic matter (SOM), total nitrogen (TN), available phosphorus (AP) and available potassium (AK). The remaining subsample was stored at −80 °C and delivered to Biomarker Technologies Co., Ltd., (Beijing, China) for high-throughput sequencing.

### 2.4 Plant growth and soil physicochemical properties determination

Plant height and basal stem diameter were measured using a ruler and vernier caliper, respectively. Plants were then divided into aboveground shoots and underground roots. Root samples were fully rinsed with clean water and scanned via STD4800 root scanner (Regent Instruments Inc., Canada). Total root length was quantified using WinRHIZO 2013 image analysis software. Shoots and roots were oven-dried at 75 °C to constant weight for biomass determination.

Soil pH and EC were determined using a pHS-4 acidimeter and DDS-11A conductivity meter with soil-water ratio of 1:5 (Guo et al., 2024). Soil organic matter (SOM) was determined using the potassium dichromate volumetric method (Guo et al., 2024), total nitrogen (TN) was determined using the Kjeldahl method (Wang et al., 2021), available phosphorus (AP) was determined by 0.5 mol/L sodium bicarbonate extraction-molybdenum antimony colorimetric method, available potassium (AK) was determined by ammonium acetate extraction-flame photometer method (Guo et al., 2024).

### 2.5 Microbial amplicon sequencing

DNA of each sample was extracted with the TGuide S96 Magnetic Soil/Stool DNA Kit [Tiangen Biotech (Beijing) Co., Ltd., (Beijing, China)] according to the manufacturer’s instructions, and the DNA concentration was measured with the Qubit dsDNA HS Assay Kit and Qubit 4.0 Fluorometer (Invitrogen, Thermo Fisher Scientific, Hillsboro, OR, USA). Fungal ITS region was amplified using the primer pair ITS1 (5′-CTTGGTCATTTAGAGGAAGTAA-3′) and ITS2 (5′-GCTGCGTTCTTCATCGATGC-3′) (Yang et al., 2017), the thermal cycling conditions were 95 °C for 5 min, 30 cycles of 95 °C for 1 min, 50 °C for 1 min, and 72 °C for 1 min, followed by 72 °C for 7 min. The bacterial 16S rRNA gene V3–V4 region was amplified using the primer pair 338 F (5′-ACTCCTACGGGAGGCAGCA-3′) and 806 R (5′-GGACTACHVGGGTWTCTAAT-3′) (Wang et al., 2020), the thermal cycling conditions were pre-denaturation at 98 °C for 2 min, denaturation at 98 °C for 15 s, annealing at 55 °C for 30 s, extension at 72 °C for 30 s, and final extension at 72 °C for 5 min (30 cycles). The PCR amplicons were gel-purified using Agencourt AMPure XP Beads (Beckman Coulter, Indianapolis, IN, USA), resultant PCR products were combined at equimolar concentrations and sequenced with Illumina NovaSeq 6000 (Illumina, Santiago CA, United States) in Beijing Biomarker Technologies Co., Ltd.

### 2.6 Data processing and analysis

Raw reads were filtered with Trimmomatic (version 0.33) and identified, primer sequences were removed with cutadapt (version 1.9.1), and then denoised with QIIME2 (version 2020.6) to obtain clean reads. Operational taxonomic units (OTUs) were clustered with 97% similarity using VSEARCH (Rognes et al., 2016). Taxonomic classification of bacterial sequences was performed against the SILVA database (v138) using the Naive Bayesian classifier implemented in QIIME2 with a confidence threshold of 70%. For fungal ITS sequences, taxonomic assignment was performed against the UNITE database (v8.2) (Nilsson et al., 2019) using the same classifier. Chao1 index were calculated and displayed by the QIIME2 and mothur (ggplot2 package, version 3.1.1; ggpubr, v1.22.2), beta diversity was analyzed by QIIME2 software (version 2020.6) with partial least squares discriminant analysis (PLS-DA), biomarkers were identified with Linear discriminant analysis (LDA) effect size (LEfSe) using Python 3, the analysis results of Mantel test heatmap plots were generated using the R software packages “vegan” through the CNSknowall^1^ a comprehensive web service for biomedical data analysis and visualization (Calculated by pearson). All statistical analyses were performed using R (version 4.5.2) and Excel 2019. Tukey’s HSD test was used for all multiple-group comparisons. Figures were generated using Origin 2021.

## 3 Results

### 3.1 Effect of SynComs on agronomic traits and stress-resistance enzyme activities in potato

SynCom inoculation significantly affected the plant height, stem diameter, root length and biomass of the potato plants (Figure 1). Compared with the control (CK), SB markedly increased the plant height at the seedling stage, and the root length at the flowering stage. SF substantially increased the total biomass and root biomass at the seedling stage, as well as the root length at the flowering stage. The SBF consortium exhibited the strongest growth-promoting effect: it increased seedling plant height, stem diameter, root length and root biomass by 16.24%, 40.84%, 25.84% and 33.18%, respectively. At the flowering stage, the plant height, root length, total biomass and aboveground biomass rose by 27.71%, 28.73%, 39.31% and 29.27%, respectively. Collectively, SynComs effectively promoted potato growth, with the SBF consortium showing the best performance. Inoculation with SynComs significantly enhanced the antioxidant enzyme activities in potato leaves and reduced the degree of membrane lipid peroxidation (Figure 2). At the S stage, compared with the CK group, the SB and SBF treatment groups showed significantly decreased malondialdehyde (MDA) content and significantly increased superoxide dismutase (SOD) activity in leaves. Meanwhile, the SBF treatment group showed significantly decreased catalase (CAT) activity. At the F stage, the SBF treatment group showed significantly reduced MDA content and significantly increased activities of peroxidase (POD), SOD, and CAT. These indicators showed that potato plants were responsive toward the different treatments, corresponding to alleviated COO stress and better growth.

**FIGURE 1:**
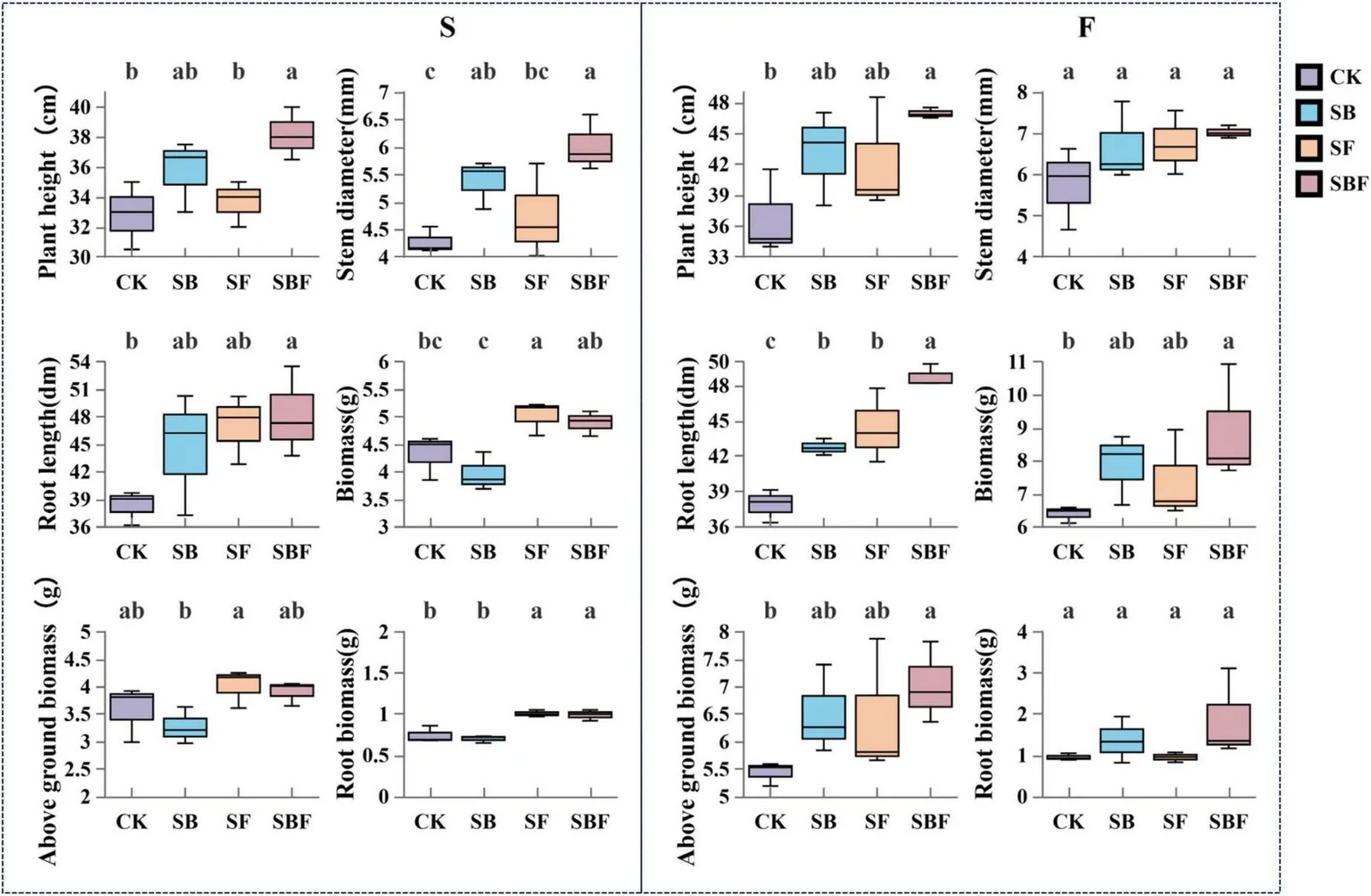
Effect of SynComs on potato plant growth. S: seedling stage; F: flowering stage. Different lowercase letters indicate significant differences between treatments when *P* < 0.05 (Tukey’s HSD multiple comparison test).

**FIGURE 2:**
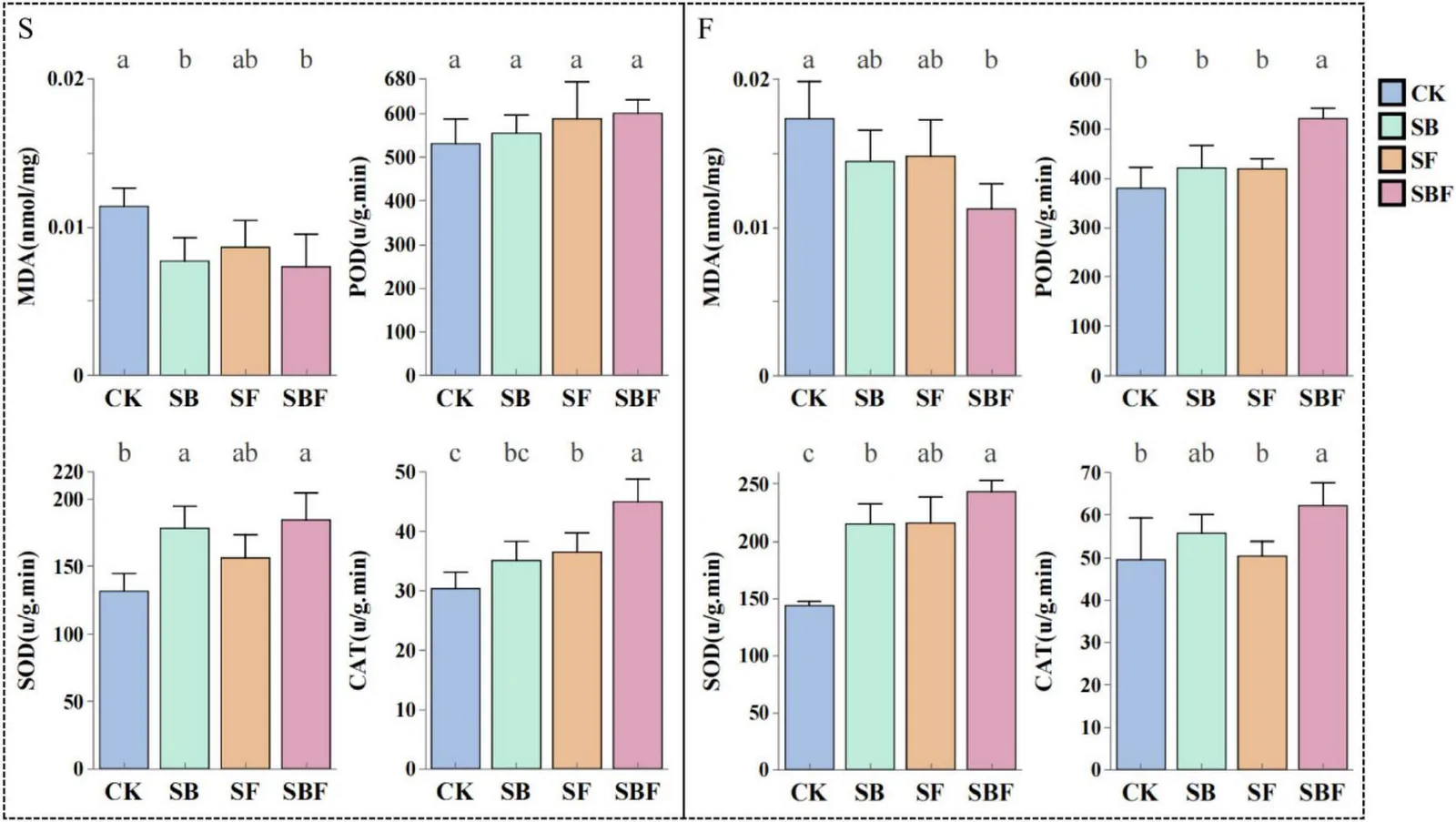
Effects of SynComs on stress-resistance enzyme activities in potato plants. S: seedling stage; F: flowering stage. Different lowercase letters indicate significant differences between treatments when *P* < 0.05 (Tukey’s HSD multiple comparison test).

### 3.2 Effect of SynComs on the soil microbial diversity

SynCom inoculation significantly changed soil microbial alpha diversity at the seedling stage (Figure 3). The SB treatment notably reduced the Chao1 indices of soil bacteria and fungi during this period. By contrast, SBF markedly increased bacterial Chao1 values at the seedling stage. At both the S and F stages, the SBF treatment exhibited the highest values of the Simpson index and the Shannon index. At the F stage, compared with the CK treatment, the SB treatment significantly decreased the Simpson index and Shannon index values.

**FIGURE 3:**
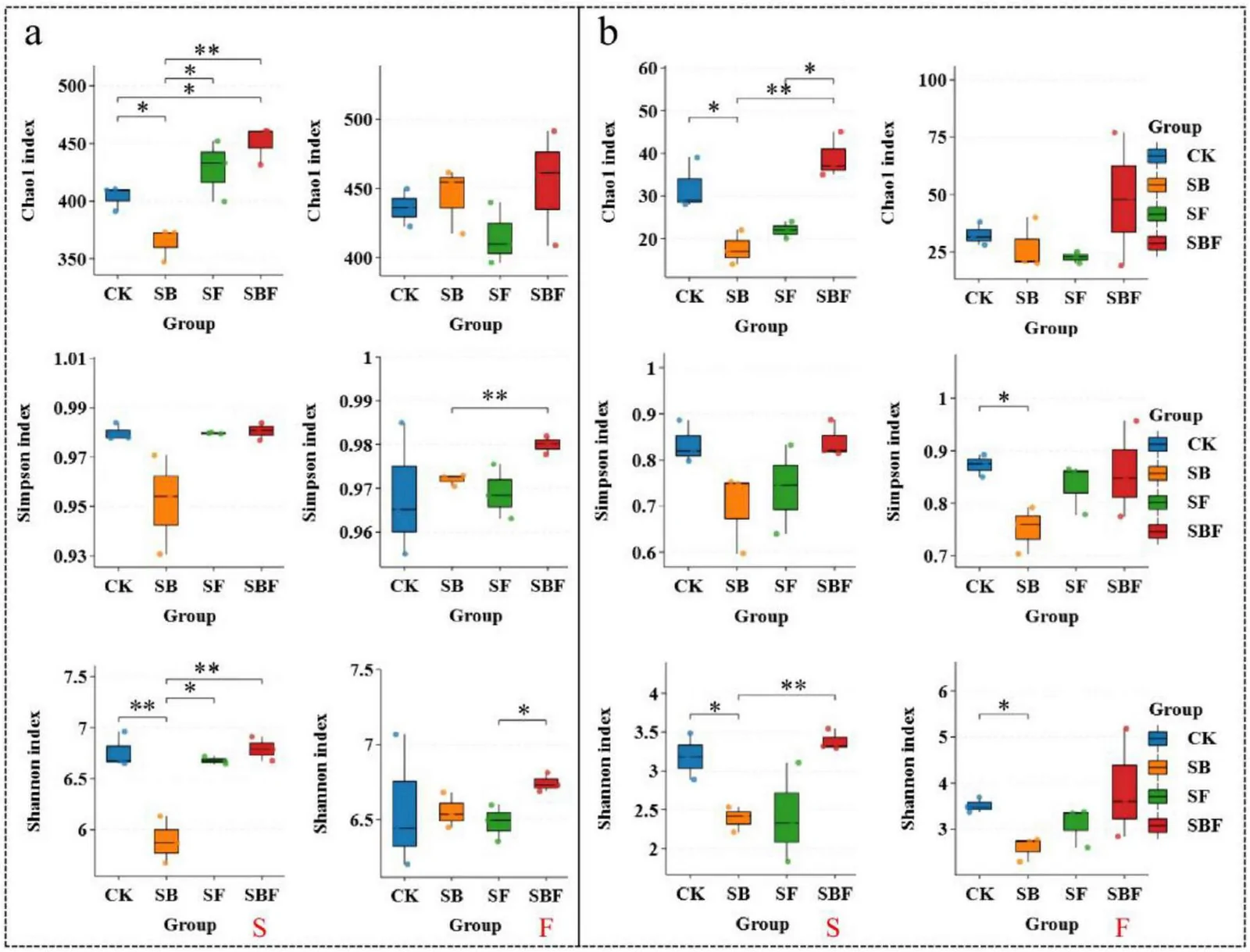
Effect of SynComs inoculation on soil microbial diversity. **(a)** Bacteria; **(b)** fungi; S: seedling period; F: flowering period. *p < 0.05, **p < 0.01.

Partial least squares discriminant analysis (PLS-DA) results demonstrated that the three SynComs substantially altered soil bacterial and fungal community compositions (Figure 4). At the seedling stage, microbial communities differed greatly between the control and inoculated groups with clear separation.

**FIGURE 4:**
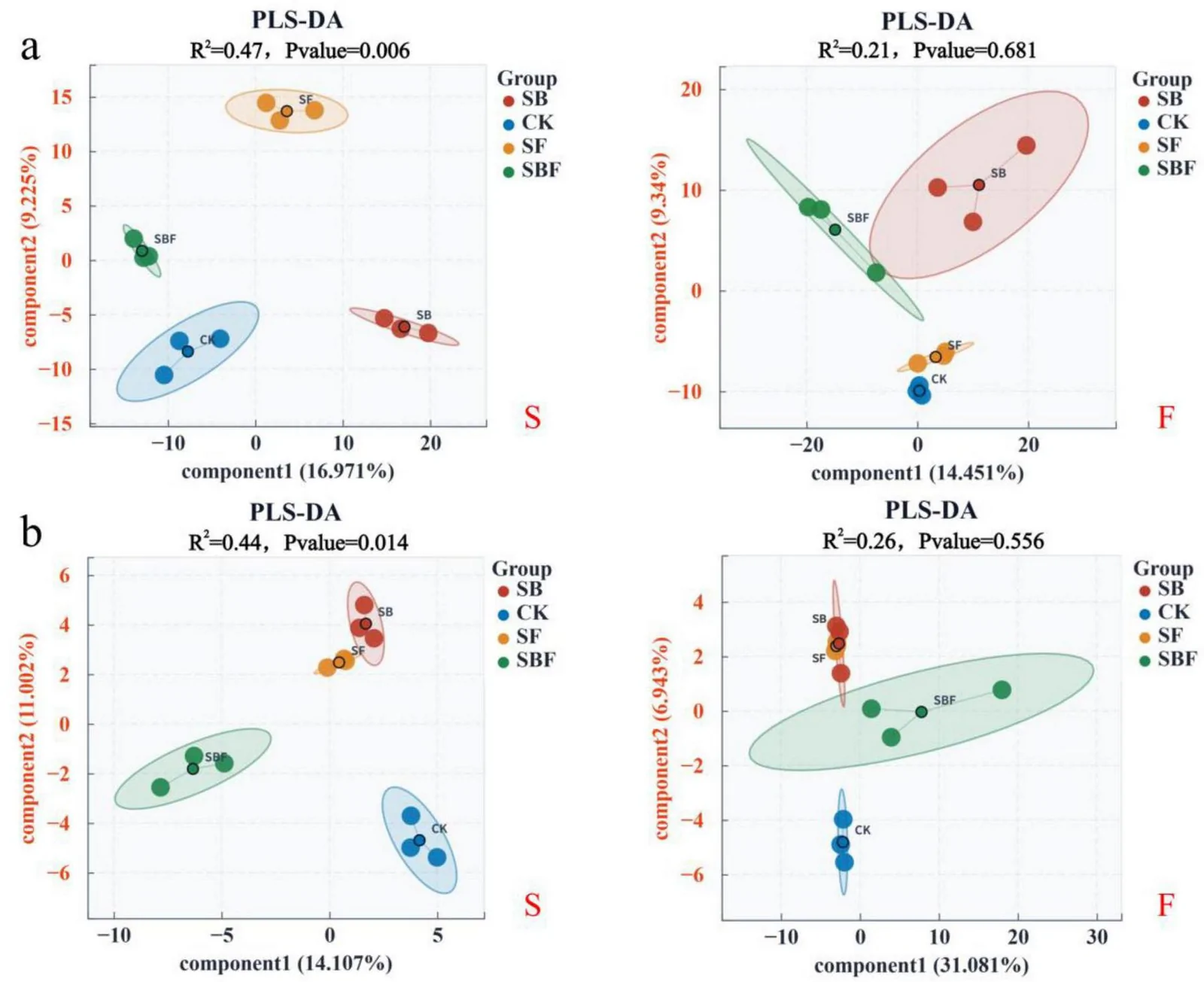
Effect of synthetic microflora application on soil beta diversity. **(a)** Bacteria; **(b)** fungi; S: seedling period; F: flowering period. The ellipses represent 95% confidence intervals.

### 3.3 Abundance dynamics of inoculate-related microbes

Analysis on the abundance of inoculate-related microbes (Figure 5) showed notable variations across treatment groups at the seedling stage. The abundances of *Pseudomonas* sp. and *Microbacterium* sp. in the SB group were significantly higher than those in other groups. Meanwhile, *Aspergillus terreus* exhibited remarkably higher relative abundance in the SBF group compared with the CK and SB groups. At the flowering stage, no obvious differences were observed in the abundance of inoculated strains across all treatment groups.

**FIGURE 5:**
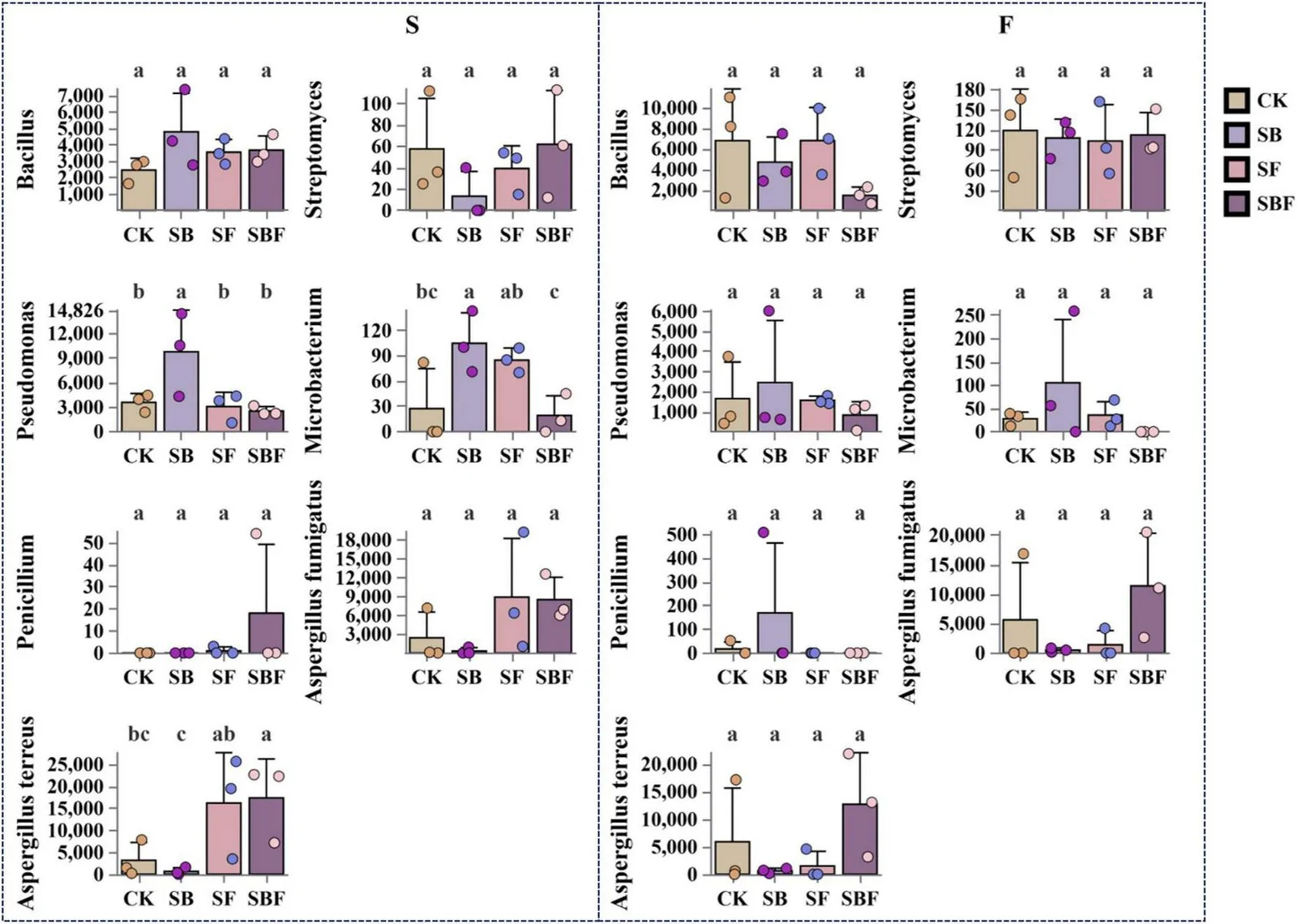
Dynamic changes in the abundance of inoculated strains under SynCom treatments. S: seedling stage; F: flowering stage. Different lowercase letters indicate significant differences between treatments when *P* < 0.05 (Tukey’s HSD multiple comparison test).

### 3.4 Effect of SynComs inoculation on the soil microbiome

Correlation network analysis indicated that dominant bacterial species remained stable across treatment groups, while fungal dominant taxa varied markedly (Figure 6). *Bacillus* dominated all treatments except seedling-stage SF and flowering-stage SBF, whereas *Pseudomonas* only predominated at the seedling stage and in flowering-stage SB. Furthermore, Unclassified Gemmatimonadaceae prevailed in SF and SBF groups, and *Sphingomonas* served as the key bacterial species in the SB group at the seedling stage, while at the flowering stage, unclassified Gemmatimonadaceae dominated all treatment groups, *Arthrobacter* was the core taxon in CK, unclassified Vicinamibacterales dominated SF and SBF groups. For fungal communities, *Talaromyces* dominated CK at the seedling stage, while *Naganishia* prevailed in CK at the flowering stage, Unclassified Pleosporales was the dominant fungal species in the SB group (Supplementary Table 2). Network analysis revealed that SB and SBF treatments reduced positive correlations among soil bacteria at the seedling stage, whereas SF treatment strengthened these correlations. In the flowering stage, SB and SBF elevated positive bacterial interactions, while SF suppressed such associations. The SBF treatment decreased positive correlations within fungal communities, and both SB and SF markedly enhanced fungal positive interactions. SynCom inoculation reshaped microbial interspecific relationships, further altering community structure and ecological functions. Network metrics showed that soil bacterial communities appeared to be less responsive to the effects of SynComs than that of fungal communities, but this observation needs further testing and confirmation. Microbial co-occurrence network topological parameters indicated that, in the bacterial networks (Supplementary Table 3), the SBF treatment at the seedling stage exhibited the highest number of nodes, along with decreased average degree of 2.50, clustering coefficient, and graph density, while displaying the highest modularity and stronger differentiation among community taxa. In contrast, the fungal networks overall showed significantly higher clustering coefficients than those of the bacterial networks, with the SB treatment exhibiting a markedly increased proportion of positive edges.

**FIGURE 6:**
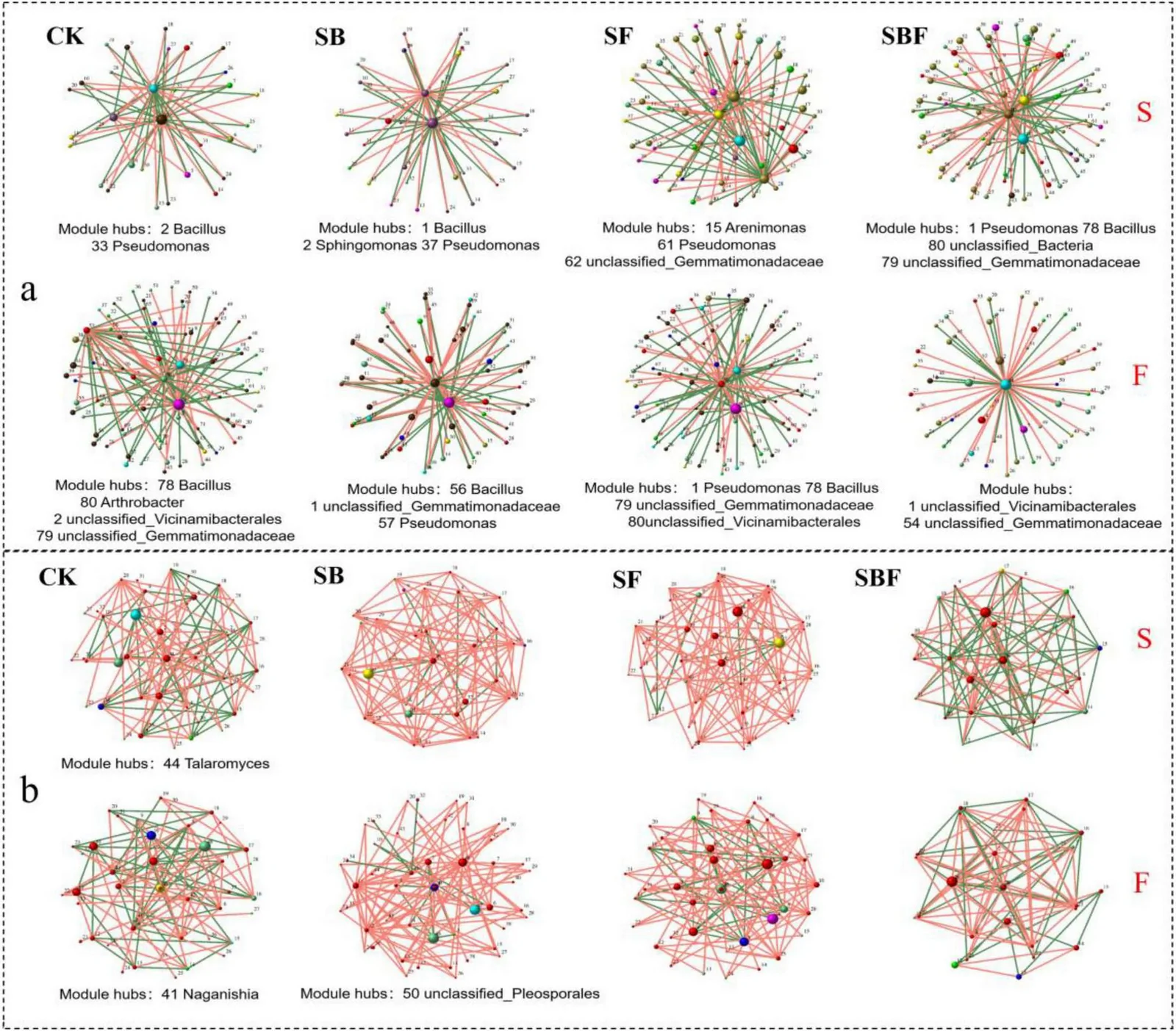
Correlation network plot between treatments. **(a)** Bacteria, **(b)** fungi; S: seedling period; F: flowering period. The correlation network was established using Spearman’s correlation analysis, focusing on genera with a relative abundance greater than 0.1%. The criteria for inclusion were a correlation coefficient (r) exceeding 0.8 and a significance level (p) below 0.05. In the visualization of the network, red lines indicate statistically significant positive associations, while green lines represent negative relationships. The genera highlighted in the figures serve as module hubs, identified based on their within-module connectivity (ZI) surpassing 2.5 and their among-module connectivity (PI) being less than 0.62.

As shown in Figure 7a, LEfSe analysis was applied to screen group-specific microbial biomarkers. Bacterial LEfSe results suggested four biomarkers in the CK group at the seedling stage and one biomarker at the flowering stage. Syncom inoculation induced the emergence of novel soil biomarkers. At the seedling stage, biomarkers including *Flavisolibacter_ginsengisoli, Gemmatimonas, Myxobacterium, Phenylobacterium* and *Sphingomonas* were detected in SB, Vicinamibacteraceae, *Arthrobacter*, Chloroflexi, *Bacillus*, Gemmatimonadaceae, Nitrospirales and *Arenimonas* in SBF, Longimicrobiaceae and *Cellvibrio* in SF. At the flowering stage, *Sphingobium* and *Marmoricola terrae* were identified in the SBF group, while *Methylophaga frappieri* served as the biomarker in the SF group.

**FIGURE 7:**
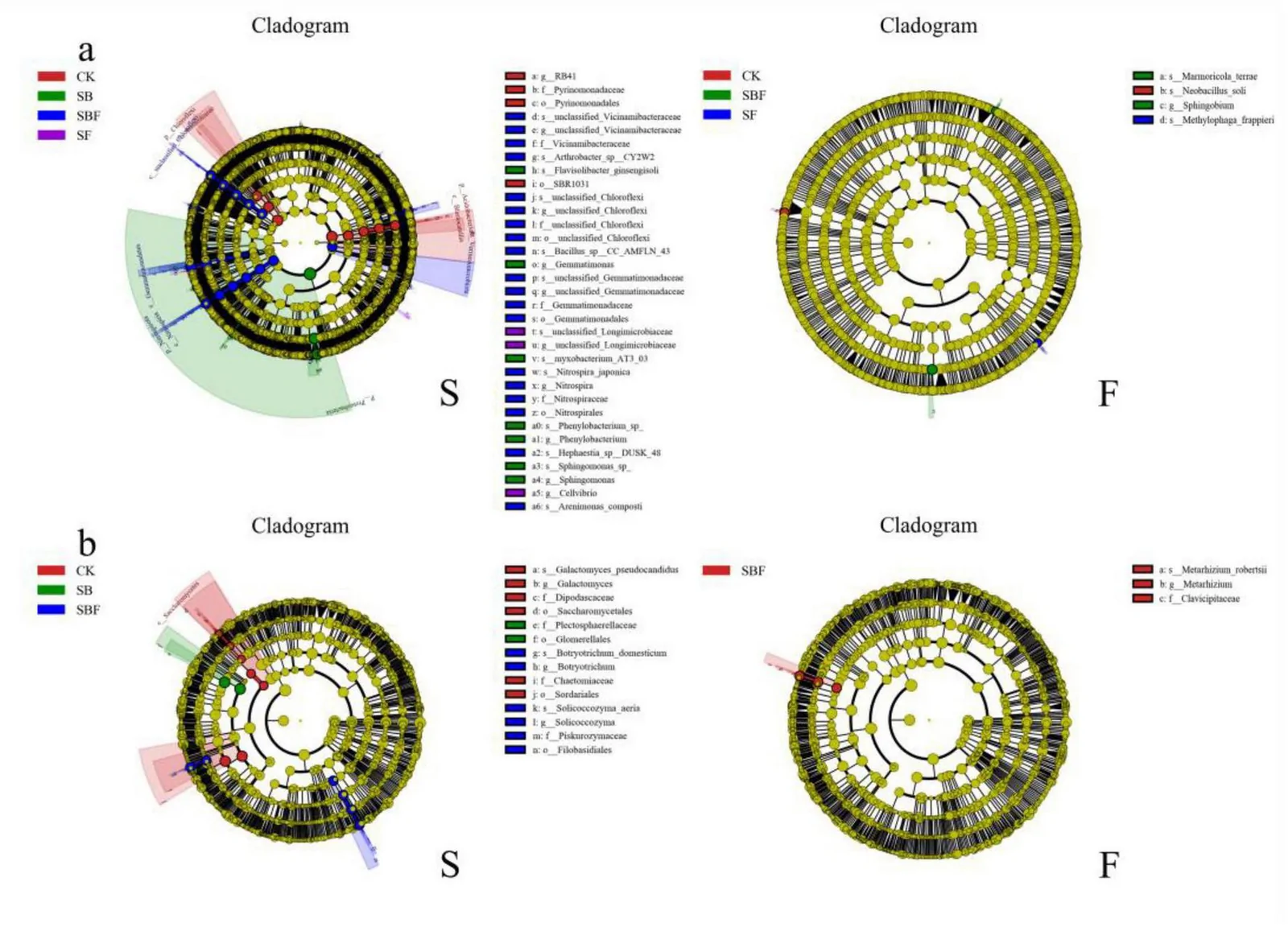
Linear discriminant analysis (LDA) effect size (LEfSe) analysis LDA > 4.0. **(a)** Bacteria; **(b)** fungi; S: seedling period; F: flowering period.

As shown in Figure 7b, LEfSe analysis of fungi identified six biomarkers in the CK group at the seedling stage. New fungal biomarkers emerged in the soil following Syncoms inoculation. The SB group had biomarkers belonging to the order of Glomerellales and the family of Plectosphaerellaceae. Biomarkers in the SBF treatment transitioned from *Solicoccozyma, Botryotrichum*, Filobasidiales, Piskurozymaceae, *Solicoccozyma aeria* and *Botryotrichum* (seedling stage) to *Metarhizium* and Clavicipitaceae (flowering stage). Functional prediction based on PICRUSt2 revealed that Metabolic pathways constituted the core dominant functional category at both growth stages (Figure 8). At the S stage, the relative abundance of the two-component system in the SB treatment group (5.19%) was significantly higher than that in the other three groups, with the SF treatment group showing the lowest value (4.68%); a similar trend of the highest abundance in the SB group was observed for Quorum sensing. The abundances of basic metabolic pathways in the SBF and SF groups were higher than those in the CK and SB groups. At the F stage, the SBF co-inoculation group exhibited the highest abundances of secondary metabolite biosynthesis (7.59%), antibiotic biosynthesis (5.71%), amino acid biosynthesis (3.29%), ribosome (1.87%), and core metabolic pathways (16.18%), whereas the SBF group had the lowest abundance of the two-component system. Meanwhile, the overall abundances of ABC transporters and the two-component system generally declined compared with those at the seedling stage. Further functional annotation via FAPROTAX indicated that chemoheterotrophy and aerobic_chemoheterotrophy were the dominant ecological functions of bacteria across all groups. Fungal functional prediction based on FUNGuild showed that, at the S stage, the SBF group exhibited increased proportions of aerobic chemoheterotrophy, ureolysis, and nitrate reduction, while the SB group displayed a marked rise in the relative abundance of plant pathogens and a decrease in the proportion of dung saprotrophs. In contrast, the SF group showed reduced relative abundance of plant pathogens. At the F stage, the SBF group showed a substantial increase in the abundance of animal pathogens, whereas the SB group showed the highest relative abundance of plant pathogens.

**FIGURE 8:**
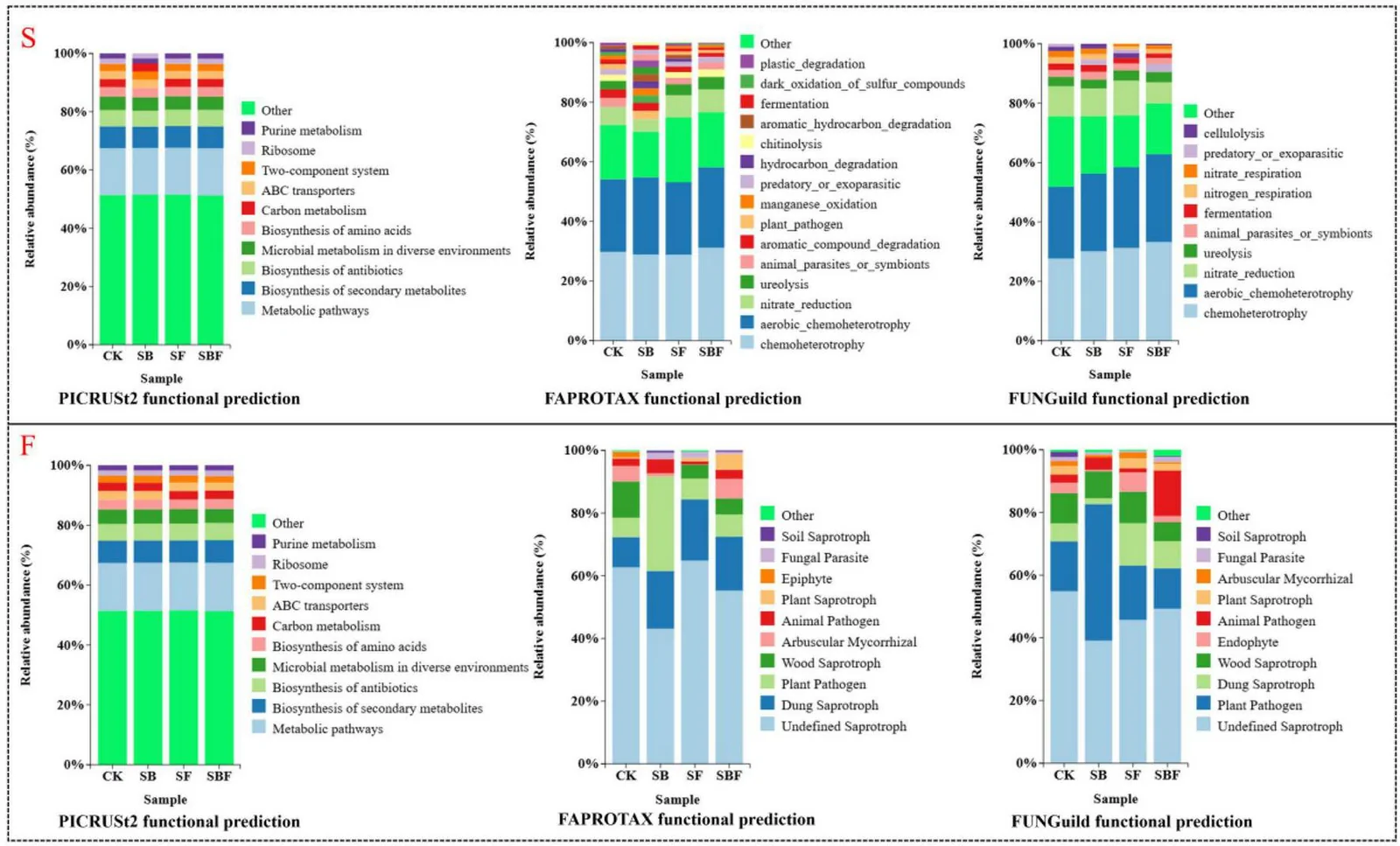
Function predictions of microbial communities (S: seedling period; F: flowering period).

### 3.5 Response relationships between environmental factors and microbial community structures

Mantel test confirmed significant correlations among soil physicochemical properties, plant growth traits and microbial communities (Figure 9). At the seedling stage, SynComs and core bacterial taxa exhibited positive correlations with the shoot biomass, the root biomass and the total plant biomass, whereas no significant correlation was detected between soil physicochemical properties and plant growth parameters. Upon transition to the flowering stage, the promoting effects of SynComs on plant growth diminished, and soil physicochemical properties began to take effect instead. Available potassium (AK) played divergent ecological roles: it was positively correlated with bacterial activity but negatively associated with plant biomass. Notably, at the F stage, the SynCom inoculation treatments significantly decreased the AP content in soil, while the SBF treatment significantly decreased the AK content and significantly increased the TN content (Supplementary Figure 1).

**FIGURE 9:**
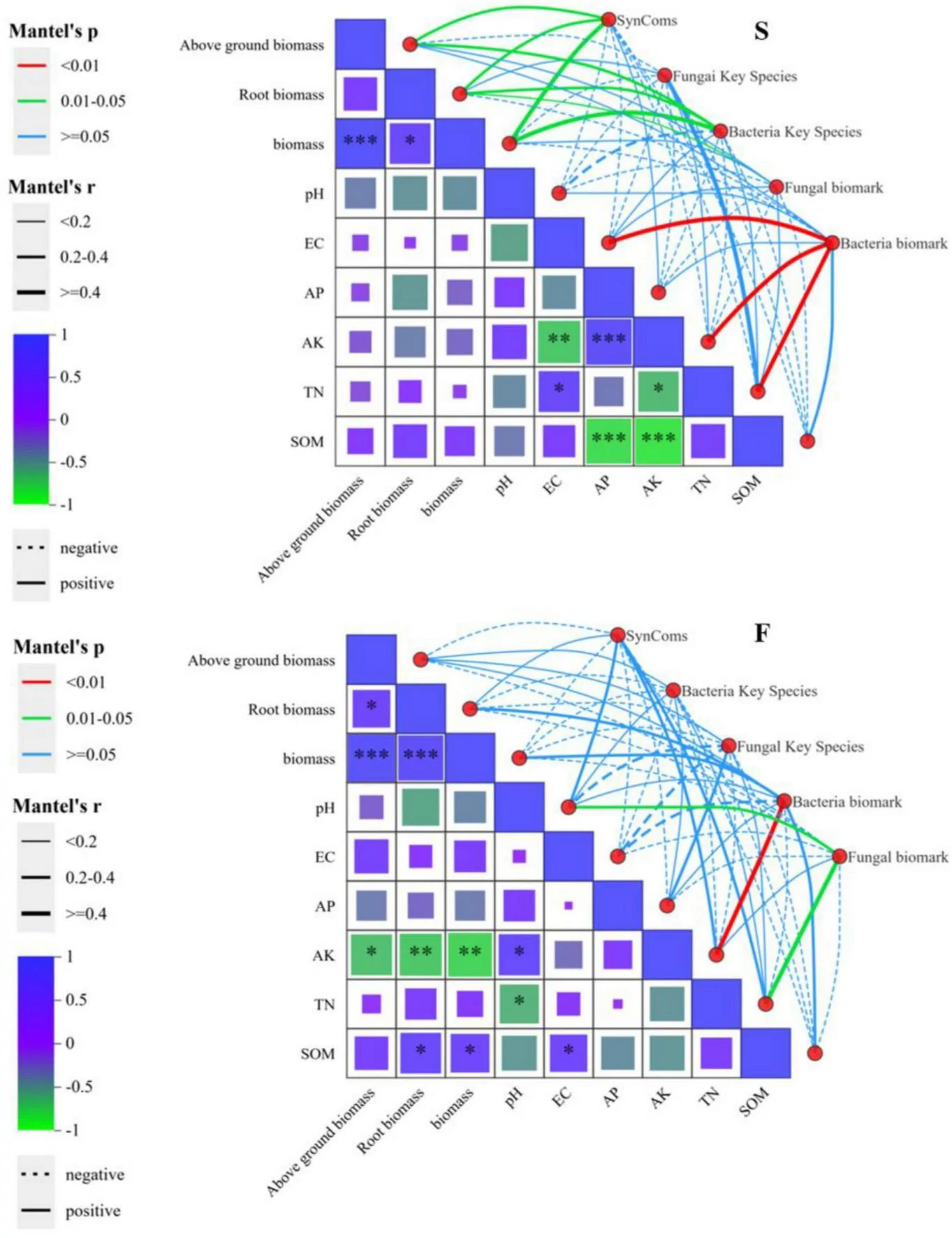
Mantel test analysis of plant growth, environmental factors, and microbial community. *, **, and *** denoted statistical significance levels of *p* < 0.05, *p* < 0.01, and *p* < 0.001, respectively. Significant positive correlations are indicated by purple squares, while negative correlations are represented by green squares. The intensity of the color and the size of the square indicate the strength of the correlation coefficient. Red lines signify *p* < 0.01, green lines indicate 0.01 < *p* < 0.05, and blue lines represent *p* ≥ 0.05. A solid line denotes a positive relationship, a dashed line represents a negative relationship, and the thickness of the line reflects the magnitude of the correlation coefficients (S: seedling period; F: flowering period).

## 4 Discussion

### 4.1 Effects of SynCom inoculation on the growth of potato plants

This study verified that SynComs improved the growth of continuously cropped potato, with the SBF consortium showing the most prominent performance, which is likely attributed to the synergistic effects within the SBF SynComs (Minty et al., 2013). During the entire growth period, SBF exerted a stronger promoting effect on the root development (Liu et al., 2023). This process increased the biomass of both above- and below-ground tissues, laying a solid material foundation for the subsequent formation and expansion of potato tubers (Wang Z. et al., 2019). It should be noted that vigorous root growth and biomass accumulation likely accelerated soil nutrient consumption, resulting in reduced soil nutrient contents. Nonetheless, SynCom application significantly changed soil physicochemical properties. Consistent with previous findings (Chen et al., 2021), SynComs could be used to optimize soil physicochemical characteristics. Notably, SynComs play dual roles in regulating soil nutrients: while they reduce nutrient levels by stimulating plant growth, they also increase nutrient availability via nutrient activation and accelerated decomposition of soil organic matter.

### 4.2 Effects of SynComs inoculation on the soil microbiome in potato cultivation

Soil microorganisms perform vital ecological functions, including decomposing soil organic matter, synthesizing humus, facilitating the transformation and cycling of soil nutrients, improving soil fertility and promoting plant growth (Cheng et al., 2023). Previous studies have demonstrated that SynCom application reshapes the composition of bacterial and fungal communities in cotton rhizosphere soil and enriches beneficial microorganisms that are crucial for restoring degraded ecosystems (Hao et al., 2023). Inoculation with SynComs was also shown to elevate the alpha diversity of soil bacteria, and to restructure the overall soil microbial community (Hao et al., 2023). However, not all effects of SynComs on soil microbiota were positive, documented decline in soil microbial diversity following the addition of SynComs with different formulations was reported as well (Kaur et al., 2022). Inoculation with a single bacterium significantly reduced alpha diversity of soil bacteria and fungi, as it could have competitive advantages over soil bacteria for growth-supporting resources. The bacteria-only SB consortium used in this study may have the same effect on soil microbiota as a single bacterium, and induce competitive exclusion among indigenous bacteria by rapidly consuming labile resources, reducing overall richness (Kaur et al., 2022). By contrast, introducing fungi in SBF creates novel microhabitats and additional carbon sources through fungal decomposition of recalcitrant polymers, accommodating specialized taxa that may not compete directly with fast-growing bacteria. Notably, the PLS-DA separation driven by SBF was strongest at the S stage, indicating that the structural reshaping of microbial communities occurs earlier than the F stage, a temporal dynamic overlooked in previous potato SynCom reports that typically sampled at a single time point (Shen et al., 2015; Zhao et al., 2018). Accordingly, inoculation with complex microbial consortia can substantially alter soil microbial community structure, improve soil physicochemical properties, and boost plant growth (De Gregorio et al., 2017). Our results also indicated that SynCom inoculation restructured soil microbial functional profiles, consistent with compositional changes. Metabolism remained the core function of the SynComs, and chemoheterotrophy and aerobic chemoheterotrophy were the dominant ecological functions across all treatments, suggesting that fundamental metabolic potential was preserved despite taxonomic shifts (Qin et al., 2023). At the flowering stage, the functional differentiation among groups attenuated, consistent with results reported by others (Nicotra et al., 2026).

Numerous studies have revealed that microbial inoculants can increase the soil microbial abundance, modify community composition, and enhance functional diversity of rhizosphere microorganisms (Poulsen et al., 2013; Peng et al., 2021). In our study, the relative abundances of beneficial phyla including Proteobacteria and Gemmatimonadota rose distinctly at the S stage, Proteobacteria, a phylum containing many well-characterized plant-growth-promoting taxa, has been widely associated with rhizosphere nutrient cycling and plant health. Gemmatimonadota has been implicated in soil carbon and nitrogen cycling in some agricultural systems (Zhang et al., 2022), though its functional roles in the potato rhizosphere require further investigation. For the genus *Bacillus*, its abundance increased in both SB and SBF groups at the S stage but declined at the F stage. *Bacillus* has been reported to enhance plant growth by modulating the composition of rhizosphere microbial communities (Shi et al., 2022). *Streptomyces* could protect host plants against pathogens across bulk soil, rhizosphere and endosphere habitats by producing antimicrobial metabolites and lytic enzymes. With the treatment of SB, the abundances of *Pseudomonas* and *Microbacterium* increased significantly. As typical plant growth-promoting rhizobacteria (PGPR), *Pseudomonas* strains are widely applied to stimulate plant growth in various cropping systems (Ha-Tran et al., 2021). Related research also showed that *Microbacterium* could promote wheat growth and remediates contaminated soils via synergistic plant-microbe interactions (Sheng et al., 2009). Fungi of the genus *Penicillium* are naturally tolerant to saline-alkali and low-temperature environments, maintaining normal metabolism and viability under such stresses (Dhakar et al., 2014). *Aspergillus terreus* evidently promotes the growth of plant stems and roots (Vassileva et al., 2020), and combined activities of *Aspergillus* and other microorganisms help improve crop yield (Hang et al., 2022). In this study, SF and SBF treatments increased the abundances of *Penicillium* and *Aspergillus* to different extents at the S stage, whereas their abundances decreased at the F stage. This trend was confirmed by the results of alpha diversity, beta diversity and Mantel test, suggesting that the effects of SynComs on soil microbes could be time-dependent, affecting the plant growth more directly at the early stage of the plant life cycle. It should be noted that the small sample size (*n* = 3) in this study may have limited the statistical power of the analyses to reveal complex patterns complicated by the inherent variability of soil microbial communities, and the conclusions need to be further confirmed in future studies.

SynCom inoculation was shown in this study to alter the microenvironment of potato rhizosphere soil in a positive way, regulate the composition and abundance of bacterial communities, and facilitate the proliferation of beneficial microbes, consistent with earlier reports (Geng et al., 2021; Ndaba et al., 2022). Correlation network and LEfSe analyses revealed that SynCom inoculation substantially altered keystone species and microbial biomarkers. *Sphingomonas* is a key bacterial genus in potato rhizosphere soil and is beneficial toward plant growth (Khan et al., 2017). *Arthrobacter* can significantly promote root growth in plants and improve the yield and quality of wheat (Bazhanov et al., 2017). *Talaromyces* is a key fungal genus that can significantly promote plant growth and biomass, thereby improving the growth and nutritional status of plants (Goh et al., 2020). *Naganishia* can adapt to extreme environmental conditions and plays a role in the cycling of soil materials (Schmidt et al., 2017). The keystone taxa identified here (e.g., *Sphingomonas, Talaromyces*) have been individually linked to plant growth promotion in previous potato studies (Khan et al., 2017; Goh et al., 2020). Critically, our network analysis provided novel evidence to show that these taxa operate as cooperative modules only in the SBF treatment group, whereas in SB treatment group they appeared to function individually, not synergistically. This modular difference, which has not been noticed in earlier research, may have contributed to the superior performance of SBF over SB. Compared to bacteria, soil fungi exhibit greater instability and sensitivity, which also contribute to the acquisition of organic nitrogen and phosphorus and the cycling of soil nutrients (Xiong et al., 2017). However, whether the observed shifts resulted from the proliferation of indigenous taxa or the successful colonization of inoculated strains remained unresolved. Unlike previous studies that employed strain-specific markers, our current data did not provide ways to disentangle these two processes. To further illustrate mechanistically the roles played by each of these two processes, techniques combining strain-specific qPCR, stable isotope probing (SIP), and fluorescent labeling should be utilized in future studies to provide direct evidence for the relative contribution of each process.

The superior performance of the SBF over single-kingdom treatments likely reflects cross-kingdom niche complementarity, wherein bacterial members rapidly utilize labile substrates while fungal partners degrade recalcitrant polymers, collectively expanding the functional repertoire available to the host plant (Minty et al., 2013). This growth-promoting magnitude notably exceeds most previously reported single-strain or binary inoculants in potato systems (Chen et al., 2022; Shi et al., 2022). Nevertheless, caution should be exercised regarding the agricultural use of *Aspergillus fumigatus*, an opportunistic human pathogen. Avirulent substitutes or strain attenuation should be considered in future studies.

## 5 Conclusion

We conducted pot experiments to assess the influences of three synthetic microbial communities on potato growth under continuous cropping conditions. SBF inoculation greatly increased key growth parameters including the plant height, stem diameter, root length, root dry weight and total biomass relative to the control. SynCom addition reshaped the diversity and composition of soil bacterial and fungal communities, with clear temporal variations. Mantel tests confirmed that keystone bacteria within the SynComs drove potato growth enhancement, especially, SBF inoculation greatly increased key growth parameters including the plant height, stem diameter, root length, root dry weight and total biomass relative to the control, yet whether the observed growth enhancement will translate to tuber yield needs to be confirmed in future studies. SynCom addition reshaped the diversity and composition of soil bacterial and fungal communities, with clear temporal variations. Mantel tests confirmed that keystone bacteria within the SynComs drove potato growth enhancement. While these microbial consortia effectively modulated soil microbiota and boosted plant growth, further optimization of the mixing ratio and multi-site field trials across diverse soil types are needed to fully validate their efficacy and broader applicability. In addition, the constraints of pot-based experiments (confined rooting volume, absence of natural climatic variation, lack of indigenous microbial reservoir from deeper soil horizons) are undeniable, which need to be addressed in future field studies to confirm the applicability of the SynComs for field conditions.

## Data Availability

The data presented in the study are deposited in the NCBI Sequence Read Archive (SRA) under BioProject accession number PRJNA1472852.

## Footnotes

1
https://cnsknowall.com
