# Supplementary material for: Cross-kingdom synthetic microbial communities outperform single-kingdom consortia in promoting potato growth under continuous cropping
Source: Front Microbiol. 2026 Sep 23;17:1920336. doi: 10.3389/fmicb.2026.1920336 (PMC13642697; doi:10.3389/fmicb.2026.1920336)
Supplement: Supplementary file 1 [file Data_Sheet_1.PDF]

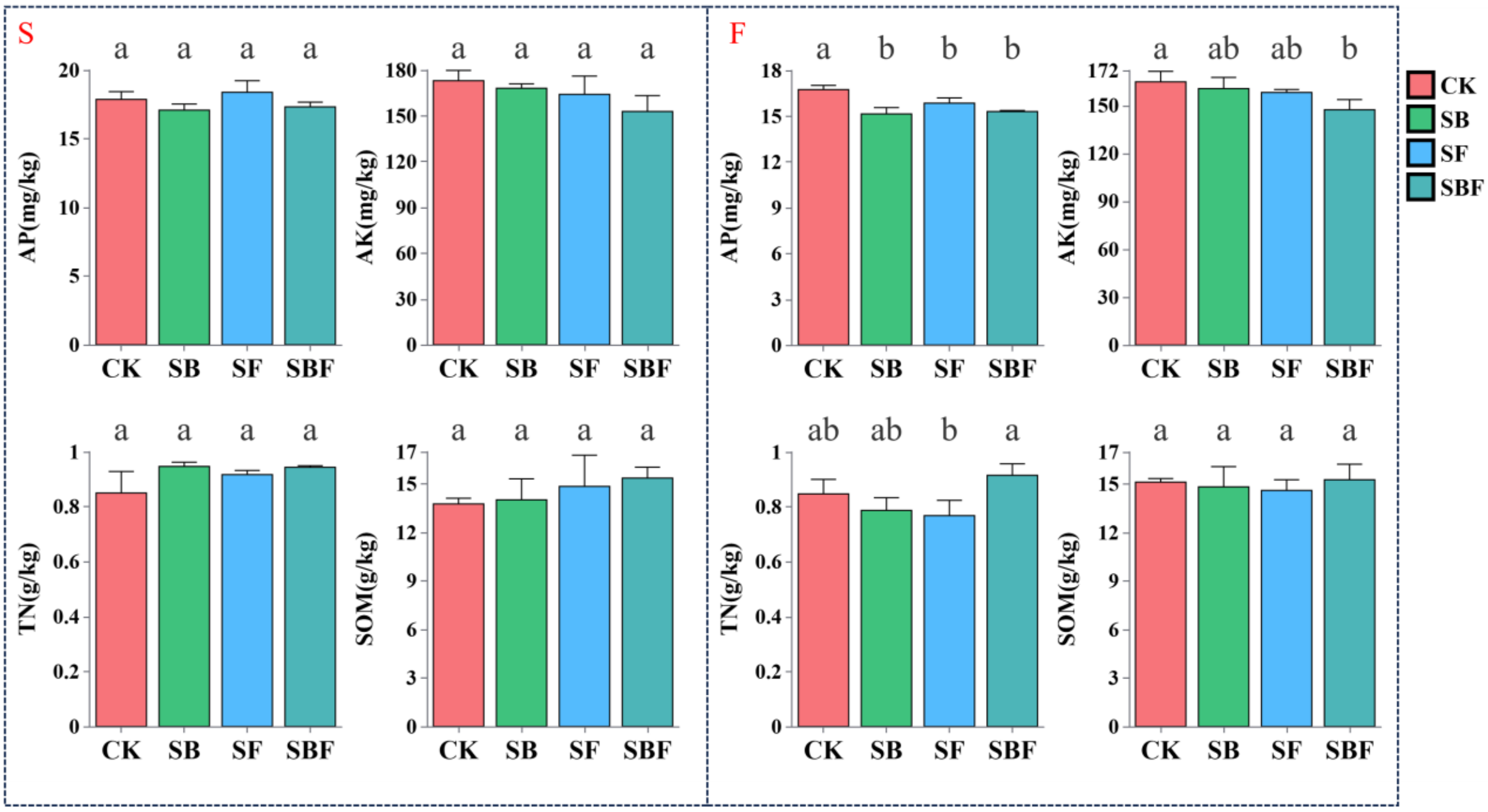

Fig. S1 Effects of SynComs Inoculation on Soil Physicochemical Properties. S: Seedling stage; F: Flowering stage. Different lowercase letters indicate significant differences between treatments when *P*<0.05 (Tukey's HSD multiple comparison test).

Table S1 Functions and physiological and biochemical characteristics of four bacterial strains capable of degrading allelochemicals

Strain	*Bacillus licheniformis*	*Streptomyces sp.*	*Pseudomonas lundensis*	*Microbacterium sp.*
NCBI GenBank accession No.	PP474459	PP474457	PP474458	PP470789
Optimal growth temperature	37 ℃	18 ℃	45 ℃	45 ℃
Optimal pH	8.0	7.0	8.0	7.0
Function	Degradation of matrine	Degradation of salicylic acid	Degradation of sinapic acid	Degradation of salicylic acid
Optimal allelochemical concentration	0.6 g/L	0.6 g/L	0.8 g/L	0.4 g/L
Degradation efficiency of 7 days	18.11%	56.87%	50.95%	37.47%
Colony characteristics	Pink, convex surface, opaque	Central convex, opaque	White, smooth surface, transparent	Yellow, smooth surface, transparent
Gram staining	Positive	Positive	Negative	Positive
Voges-Proskauer test	Positive	Negative	Negative	Negative
Indole test	Positive	Positive	Positive	Negative
Citrate utilization test	Positive	Negative	Positive	Negative
Gelatin liquefaction test	Negative	Positive	Negative	Negative
Lipase test	Negative	Negative	Negative	Negative
Glycolysis test	Positive	Positive	Negative	Positive

Table S2 Dominant microbial groups under correlation network analysis.

	CK	SB	SF	SBF
a-S	Bacillus<br>Pseudomonas	Bacillus<br>Pseudomonas<br>Sphingomonas	Arenimonas<br>Pseudomonas<br>unclassified_<br>Gemmatimonadaceae	Pseudomonas<br>Bacillus<br>unclassified_<br>Gemmatimonadaceae<br>unclassified_Bacteria
a-F	Bacillus<br>Arthrobacter<br>unclassified_<br>Gemmatimonadaceae<br>unclassified_<br>Vicinamibacterales	Bacillus<br>Pseudomonas<br>unclassified_<br>Gemmatimonadaceae	Pseudomonas<br>Bacillus<br>unclassified_<br>Gemmatimonadaceae<br>unclassified_<br>Vicinamibacterales	unclassified_<br>Vicinamibacterales<br>unclassified_<br>Gemmatimonadaceae
b-S	Talaromyces	no	no	no
b-F	Naganishia	unclassified_Pleosporales	no	no

Note: a: Bacteria, b: Fungi; S: Seedling period; F: Flowering period.

Table S3 Basic topological properties of the network.

	Treatment	Nodes	Average degree	Clustering coefficient	Graph density	modularity	Positive edges	Negative edges
a-S	CK	33	6.06	0.20	0.19	0.055	47	53
	SB	38	5.26	0.15	0.14	0.03	39	61
	SF	62	3.23	0.11	0.05	0.58	52	48
	SBF	80	2.50	0.06	0.03	0.66	48	51
b-S	CK	44	4.55	0.76	0.11	0.75	62	38
	SB	36	5.56	0.95	0.16	0.66	89	11
	SF	44	4.55	0.77	0.11	0.77	88	12
	SBF	27	7.41	0.74	0.28	0.51	48	52
a-S	CK	80	2.5	0.05	0.03	0.61	46	54
	SB	57	3.51	0.06	0.06	0.16	52	48
	SF	80	2.50	0.06	0.03	0.67	46	54
	SBF	53	3.77	0.06	0.07	0.01	63	37
b-F	CK	43	4.65	0.81	0.11	0.72	57	43
	SB	51	3.92	0.28	0.08	0.54	86	14
	SF	39	5.13	0.75	0.13	0.71	76	24
	SBF	27	7.41	0.86	0.28	0.54	58	42

Note: a: Bacteria, b: Fungi; S: Seedling period; F: Flowering period.
